# A case of treating polypoidal choroidal vasculopathy subretinal fluid by subthreshold micropulse laser

**DOI:** 10.1016/j.ajoc.2024.102225

**Published:** 2024-11-26

**Authors:** Sarmad Mustafa Jafar, Zaid Rajab Hussein, Muthanna Basheer Yasir

**Affiliations:** a Jenna Ophthalmic Center, Baghdad, Iraq; b Iraqi Ministry of Health, Ibn Al Haitham Teaching Eye Hospital, Baghdad, Iraq

**Keywords:** Polypoidal choroidal vasculopathy, Micropulse laser, Subretinal fluid

## Abstract

**Purpose:**

Assess the effectiveness of a subthreshold micropulse laser for treating a patient with polypoidal choroidal vasculopathy and subretinal fluid.

**Observations:**

A 55-year-old female presented with left eye blurring vision and metamorphopsia, and her visual acuity was 20/60. Optical coherence tomography (OCT) and OCT angiography showed subretinal fluid and pigmented epithelium detachment with a small polyp under the retinal pigmented epithelium, which was diagnosed as polypoidal choroidal vasculopathy. She was treated with a subthreshold micropulse (577nm) laser and showed a dramatic response within 12 weeks with the disappearance of subretinal fluid and visual acuity improving to 20/25.

**Conclusion and importance:**

A subthreshold micropulse laser might be an effective and safe option for treating patients with polypoidal choroidal vasculopathy and subretinal fluid.

## 1 Introduction

Polypoidal choroidal vasculopathy (PCV), or polypoidal choroidopathy, is a disease of the choroidal vasculature, present in both men and women of many ethnicities, characterized by serosanguineous detachments of the pigmented epithelium and exudative changes. It was first described by L. Yannuzzi.^1^

Polypoidal choroidal vasculopathy is most commonly diagnosed in patients between the ages of 50 and 65 years, and in many Asian countries, researchers have estimated the prevalence of PCV among their population; in Japan, higher prevalence rates are present at approximately 23–54 % in patients with presumed age-related macular degeneration (AMD).^2^ Prevalence rates of between 4 % and 9.8 % have been reported in Caucasian patients with presumed AMD.^3^

A thorough history and dilated fundus exam are the first steps in diagnosis. On fundus examinations, one can see orange-reddish bulb-like lesions budding from the choroid into the subretinal space. These lesions can be associated with recurrent and significant hemorrhagic and/or exudative detachments of the retina and retinal pigment epithelium (RPE), and subtle subretinal fluid and hard exudates are also common. On occasion, patients may present with breakthrough vitreous hemorrhage.^4^

Imaging modalities used for confirmation of the diagnosis of PCV include Indocyanine Green Angiography (ICGA), Fluorescein Angiography (FA), and high-resolution optical coherence tomography. OCT assists in making the diagnosis of this clinical entity. Although ICGA is currently considered the gold standard for diagnosing PCV, it is invasive, costly, and time-consuming, and access can be limited in some countries. Therefore, the use of simple, fast, and easily available non-ICGA imaging methods for diagnosing PCV has significant clinical benefits. The use of OCT appears to be able to diagnose PCV with the greatest accuracy, but the addition of other modalities could serve as confirmatory measures. Accordingly, the use of diagnostic features based on both OCT images and clinical features has been shown in multiple studies to be very valuable in distinguishing PCV from typical neovascular age-related macular degeneration.^5^

Several studies have evaluated treatment for PCV. Treatment includes observation, photodynamic therapy, intravitreal injection of anti-VEGF therapy, or combination therapy. The EVEREST trial^6^ is a multi-center, double-masked trial that compared three treatment regimens: verteporfin photodynamic therapy (PDT) plus the anti-VEGF agent ranibizumab (Lucentis), ranibizumab monotherapy, and PDT monotherapy. PDT in combination with ranibizumab and PDT monotherapy showed a significantly higher proportion of patients with complete polyp regression at month 6 than the ranibizumab monotherapy group. PLANET study^7^ found that at 12 months, aflibercept monotherapy was non-inferior to aflibercept with PDT in visual outcome. EPIC study^8^ at 6 months of aflibercept monotherapy stabilized vision and resulted in the resolution of hemorrhagic and exudative complications and the regression of polyps in around 70 % of patients.

## 2 Case report

A fifty-five-year-old female presented to Jenna-Ophthalmic Center in Baghdad complaining of blurring vision of the left eye for three weeks duration with metamorphopsia and a medical history of a chronic renal problem that was diagnosed as renal insufficiency syndrome with hypertension and on medical treatment of Captopril tablets 25 mg twice daily with an unremarkable past ocular history.

On clinical examination, the right eye was normal with a visual acuity was 20/20, while the visual acuity of the left eye was 20/60 corrected to 20/40 with +1.0 with a clear cornea, and an intraocular pressure was 15 mmHg and a clear crystalline lens. Fundus examination showed bilateral small drusens with loss of foveal depression in the left macula.

**Fig. 1:**
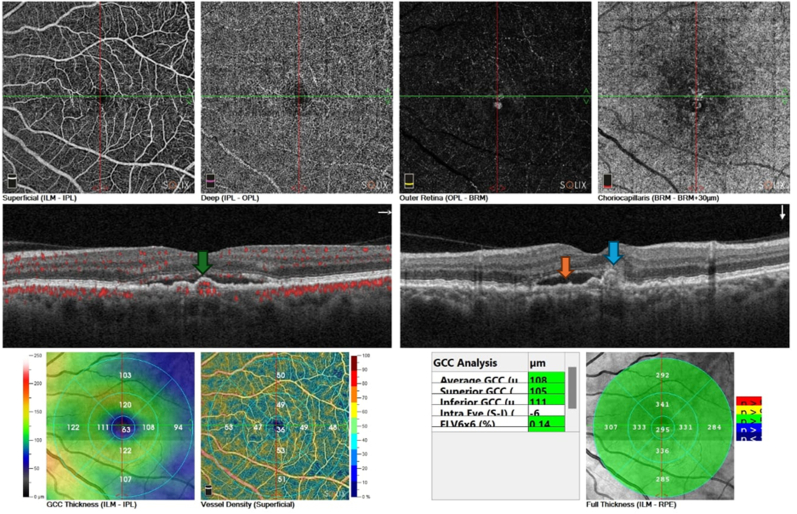
Left eye OCT and OCT angiography at presentation. These Show choroidal polyp (Blue arrow), Subretinal fluid (Orange arrow), Choroidal polyp with active flow by OCTA (Green arrow).

OCT and OCT-angiography were performed on both eyes using the Optovue Solix by Visionix (Visionix, Inc.,Fremont,CA,USA), showing that the left eye had subretinal fluid and a small, notched pigmented epithelium detachment with active flow inside it which highly suggests a choroidal polyp as shown in Fig. 1.

**Fig. 2:**
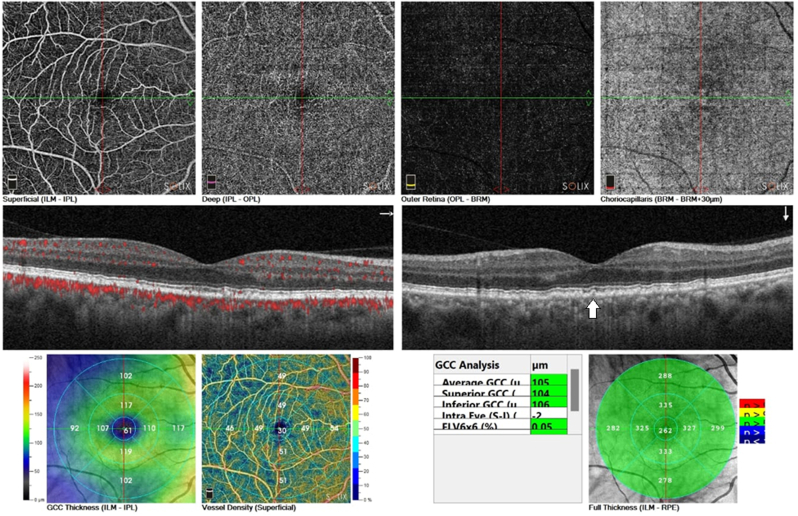
OCT and OCT angiography of the right eye at presentation, showing normal OCT except small drusen(arrow).

While the right eye OCT and OCT angiography show normal fundus except a few small drusen as shown in Fig. 2.

Fluorescein angiography couldn't be done due to renal problems, and Indocyanine green angiography (ICG) was not available in Iraq; therefore, the diagnosis was presumed to be polypoidal choroidal vasculopathy according to clinical features with OCT and OCT angiography findings.

Treatment options were discussed with the patient, and repeated anti-VEGF (aflibercept) was the first choice of treatment, but the patient refused intravitreal injection and asked for another modality of treatment without injection. Because there was no photodynamic therapy available in Iraq, a subthreshold micropulse laser was offered to her, and it was discussed that this was a new treatment modality. She accepted it and was done on the second day of the presentation using the IQ 577 laser system from IRIDEX Corporation (Mountain View,CA,USA) with the following settings and zero space: Duty cycle 5 %, power: 400 MW, size = 200 μm, duration: 200 ms.

**Fig. 3:**
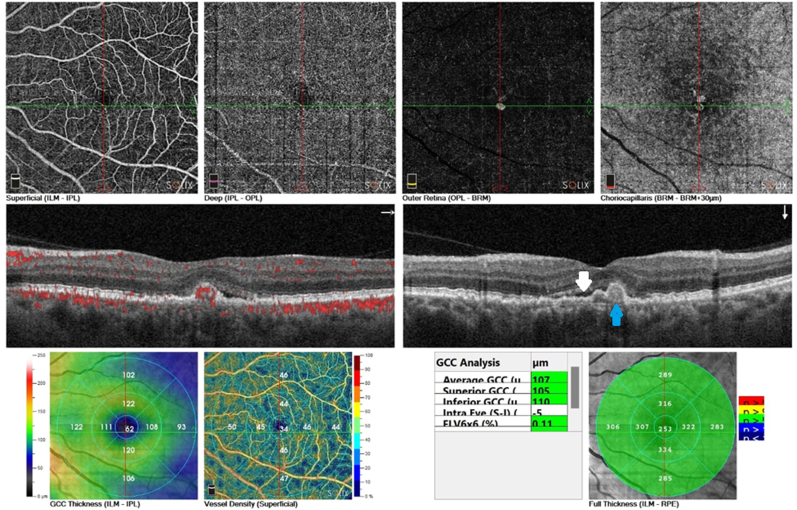
Left eye OCT and OCT angiography after 4 weeks of treatment by micropulse, showing decrease of subretinal fluid (white arrow), choroidal polyp (blue arrow).

The patient was reexamined 4 weeks later with subjective improvement in vision and a decrease in metamorphopsia, and the visual acuity was 20/30 in the left eye with a decrease in subretinal fluid in OCT, as shown in Fig. 3.

**Fig. 4:**
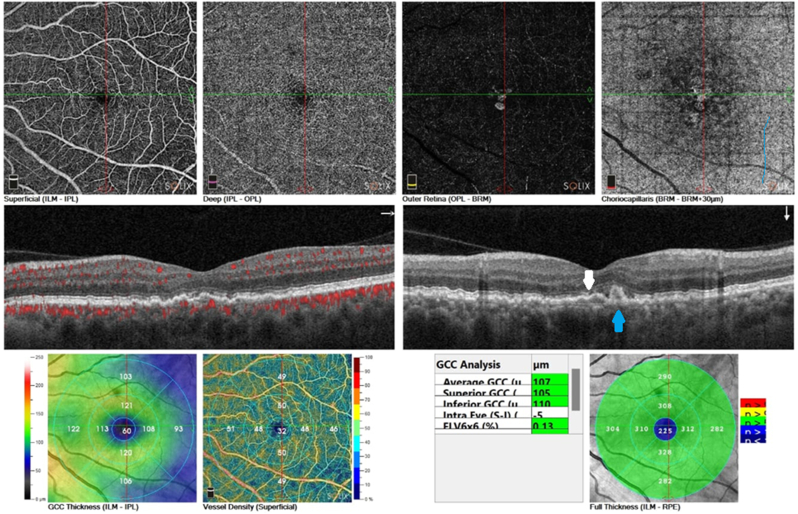
Left eye OCT and OCT angiography after 12 weeks of treatment by micropulse, showing no subretinal fluid (white arrow), choroidal polyp (blue arrow).

The patient had continuous improvement with time and, after 12 weeks, had no metamorphopsia and a visual acuity was 20/25. OCT and OCT angiography showed complete dryness of the macula with no subretinal fluid, as shown in Fig. 4.

**Fig. 5:**
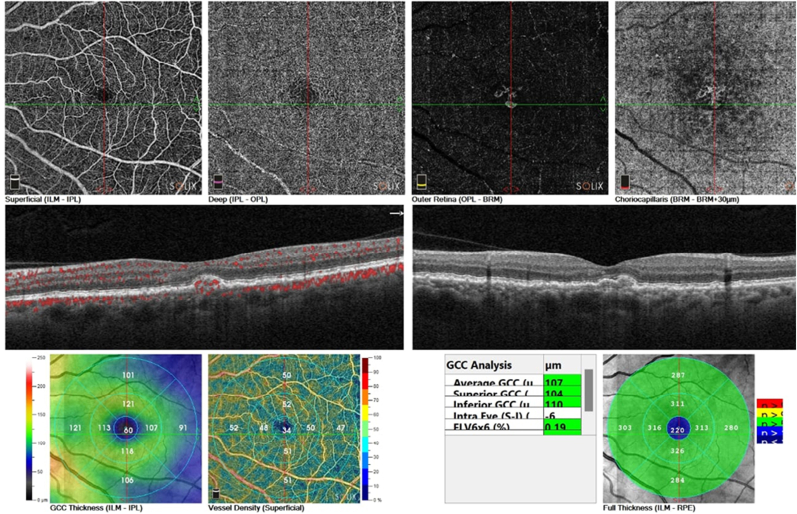
Left eye OCT and OCT angiography after 24 weeks of treatment by micropulse, showing no subretinal fluid.

The patient continued follow-up, and the vision remained stable on 20/25, and OCT and OCT angiography were stable with no signs of reactivity after 24 weeks of treatment, as shown in Fig. 5.

## 3 Discussion

In the 1990s, IRIDEX micropulse 810 nm and later 577 nm laser emitted bursts of short, repetitive pulses that last for microseconds, allowing for significant cooling in between these short pulses.^9^ This contrasts with conventional continuous wave laser, in which energy delivered continuously for milliseconds causes thermal damage. This laser emission allows for effective subthreshold tissue-sparing laser treatments without visible burns to the RPE. For the micropulse system, the ON-time (typically 100μs–300μs) is the duration of each micropulse, and the OFF-time (generally 1,700μs-1,900μs) is the interval between consecutive micropulses; therefor short ON-time limits thermal elevation and thermal dissipation to the adjacent tissue, and long OFF-time allows the tissue to cool down before the next pulse and allows the retinal pigmented epithelium RPE to return to its baseline temperature before the start of the next pulse. The low-duty cycle eliminates cumulative or continuous thermal buildup allowing for heat dissipation and heat transfer to adjacent tissue, decreasing collateral damage to the neurosensory retina, and preventing coagulative necrosis.^9^

Numerous studies^10^ confirm the safety of subthreshold micropulse laser treatment with no detectable damage to retinal pigmented epithelium, RPE or photoreceptors. Laser power is set at a low level so that the laser impact does not leave any traces on the retina. Consequently, only a limited thermal impact is exerted on the tissue, without any lethal effect.

Subthreshold micropulse laser has been tried with different results in the treatment of central serous chorioretinopathy, diabetic macular edema, proliferative diabetic retinopathy (PDR), and macular edema secondary to retinal vein occlusion.^11^

There is only one report of using subthreshold micropulse laser for treating polypoidal choroidopathy,^12^ in which the micropulse laser was used for treating a 61-year-old male patient with subretinal fluid due to PCV and showed that it was effective in decreasing the subretinal fluid after 12 weeks. It was found that the fluid completely regressed after 6 months, and in the 22nd month, subretinal fluid disappeared completely and PED appearance was observed. This is compatible with our case witch also had a good response to the micropulse laser with an earlier response and the disappearance of subretinal fluid within the first four weeks.

It is believed that the main mechanism of action of a subthreshold micropulse laser is to stimulate RPE to produce heat shock proteins (HSP) and, in this way, immunomodulate its metabolism and trigger repair processes, and then normalize the production of certain cytokines, which reduces chronic inflammation that was previously present in the retina. This theory is supported by experimental research by Inagaki et al.^13^ proves the up-regulation of HSP70 in the culture of human epithelial ARPE-19 cells after the application of a micropulse laser without any thermal damage, and also shows subthreshold micropulse laser decrease VEGF levels in the retina and eye and restore Muller cell function.^14^

The treatment approach presented in this case received approval from Jenna-Ophthalmic Center ethics committee (Approval number:34) and was conducted in adherence to the principles outlined in the declaration of Helsinki.

## 4 Conclusion

A subthreshold micropulse laser might be effective for treating polypoidal choroidal vasculopathy with subretinal fluid, but a larger control study with a larger number of patients and a longer duration of follow-up is needed.

## CRediT authorship contribution statement

**Sarmad Mustafa Jafar:** Data curation, Conceptualization. **Zaid Rajab Hussein:** Writing – original draft. **Muthanna Basheer Yasir:** Validation, Resources.

## Patient consent

Consent to publish the case report was obtained. This report does not contain any personal information that could result in patient identification.

## Authorship

All authors attest that they meet the current ICMJE criteria for Authorship.

## Funding

No funding or grant support

## Declaration of competing interest

The authors declare that they have no known competing financial interests or personal relationships that could have appeared to influence the work reported in this paper.
